# Data Collection for Automatic Depression Identification in Spanish Speakers Using Deep Learning Algorithms: Protocol for a Case-Control Study

**DOI:** 10.2196/60439

**Published:** 2025-07-31

**Authors:** Luis F Brenes, Luis A Trejo, Jose Antonio Cantoral-Ceballos, Daniela Aguilar-De León, Fresia Paloma Hernández-Moreno

**Affiliations:** 1 School of Engineering and Sciences Tecnologico de Monterrey Monterrey Mexico; 2 School of Medicine and Health Sciences Tecnologico de Monterrey Monterrey Mexico

**Keywords:** automated depression diagnosis, deep learning for audio analysis, depression binary classification, depression Spanish language dataset, high-quality and smartphone audio recordings, high-quality audio data

## Abstract

**Background:**

Depression is a mental health condition that affects millions of people worldwide. Although common, it remains difficult to diagnose due to its heterogeneous symptomatology. Mental health questionnaires are currently the most used assessment method to screen depression; these, however, have a subjective nature due to their dependence on patients’ self-assessments. Researchers have been interested in finding an accurate way of identifying depression through an objective biomarker. Recent developments in neural networks and deep learning have enabled the possibility of classifying depression through the computational analysis of voice recordings. However, this approach is heavily dependent on the availability of datasets to train and test deep learning models, and these are scarce. There are also very few languages available. This study proposes a protocol for the collection of a new dataset for deep learning research on voice depression classification, featuring Spanish speakers, professional and smartphone microphones, and a high-quality recording standard.

**Objective:**

This work aims to create a high-quality voice depression dataset by recording Spanish speakers with a professional microphone and strict audio quality standards. The data are captured by a smartphone microphone as well for further research in the use of smartphone recordings for deep learning depression classification.

**Methods:**

Our methodology involves the strategic collection of depressed and nondepressed voice recordings. A minimum participation of 60 subjects was established and 2 health centers were selected to gather data. A total of 3 types of data are collected: voice recordings, depression labels (using the Patient Health Questionnaire-9), and additional data that could potentially influence speech. Recordings are captured with professional-grade and smartphone microphones simultaneously to ensure versatility and practical applicability. Several considerations and guidelines are described to ensure high audio quality and avoid potential bias in deep learning research.

**Results:**

This data collection effort immediately enables new research topics on depression classification. Some potential uses include deep learning research on Spanish speakers, an evaluation of the impact of audio quality on developing audio classification models, and an evaluation of the applicability of voice depression classification technology on smartphone apps.

**Conclusions:**

This research marks a significant step toward the objective and automated classification of depression in voice recordings. By focusing on the underrepresented demographic of Spanish speakers, the inclusion of smartphone recordings, and addressing the current data limitations in audio quality, this study lays the groundwork for future advancements in deep learning–driven mental health diagnosis.

**International Registered Report Identifier (IRRID):**

DERR1-10.2196/60439

## Introduction

### Background

Depression is a mental health disease that affects around 5% of the world’s population; nonetheless, it can go as high as 7% in some countries [1,2]. Despite being a common disease, depression goes frequently undiagnosed or misdiagnosed, preventing affected people from getting the psychological treatment they need [3]. Detecting depression can be difficult, due to a variety of reasons. First, the 2 most common depression symptoms tend to be low energy and sleeping problems, which can be easily found in individuals without depression [1]. Second, although there are many symptoms associated with depression, the patients rarely present all of them, and the combination of symptoms can vary greatly. Fried and Neese [4] (2015) found that only 1.24% of patients showed the most common combination of symptoms. Third, according to the American Psychiatric Association, it is especially difficult to evaluate depression symptoms when they occur in an individual who also has a general medical condition [5]. This is why many professionals seek to use complementary tools in the diagnostic process. Nowadays, most depression diagnosis is done through validated health questionnaires such as the Patient Health Questionnaire (PHQ-9) and the Beck Depression Inventory (BDI-II). However, these diagnostic methods are based on patient self-reports which are influenced by their attitude, expressiveness, and familiarity with the questionnaire [6]. Besides, many people who present depression report not relating to any of the symptoms presented by the questionnaires [1]. This has made researchers interested in finding an alternative and more objective method to identify depression symptoms. Recent research has shown that the computational analysis of speech recordings can be a useful way of identifying depression symptoms objectively. The best algorithms up to this date can perform binary classification with *F*_1_-scores of around 85% [7,8]. This has been made possible by the recent progress in the field of neural networks and deep learning. However, neural networks are heavily dependent on the availability of labeled training data and depression datasets are scarce [6,9].

### Related Work

The dataset we built in this work, called D3TEC (TEC de Monterrey’s Depression Detection Dataset), stands out for providing 2 new types of data previously unavailable in voice depression classification: Spanish recordings and simultaneous recordings using both professional and smartphone microphones. Moreover, audio quality standards are higher than most publicly available voice depression datasets.

As of now, most research on automated depression classification using voice recordings has been done using the Distress Analysis Interview Corpus/Wizard-of-Oz Dataset (DAIC-WOZ dataset), which contains only English-spoken recordings, and there has been very little research in other languages [6,10]. The scarcity of non-English datasets, as noted by Wu et al [6], is one of the most important roadblocks in developing speech depression classification models. The D3TEC dataset addressed this issue by providing a collection of high-quality recordings in Spanish.

This work is also the first to record participants with both a professional and a smartphone microphone simultaneously. This enables researchers to evaluate the effect of the recording device when training deep learning models for voice depression classification. As shown in Table 1, D3TEC is the only publicly available dataset to feature smartphone recordings as well. This is important as smartphone research makes technology developments much more scalable.

**Table 1:** Comparison of different datasets used for mental health disorders detection and classification, based on audio recordings and different languages.

Dataset	Language	Audio format	Microphone	Label	Size	Year
D3TEC^a^	Spanish	WAV 44.1 kHz, 24 bits	Shure SM27 (condenser mic) and iPhoneSE2020	PHQ-9^b^	62 subjects, 1674 recordings	2024
DAIC-WOZ^c^	English	WAV 16 kHz, 16 bits	Lapel mic (unspecified)	PHQ-8^d^	189 subjects, 189 recordings	2014
AVEC^e^ 2013	German	WAV 41 kHz, 16 bits	Headset mic (unspecified)	BDI-II^f^	84 subjects, 150 recordings	2013
AVEC 2014	German	AAC 128 kbps (compressed)	Headset mic (unspecified)	BDI-II	84 subjects, 300 recordings	2014
MODMA^g^	Chinese	WAV 44.1 kHz, 24 bits	Neumann TLM102	BDI-II	52 subjects, 1508 recordings	2020

^a^D3TEC: TEC de Monterrey’s Depression Detection Dataset

^b^PHQ-9: Patient Health Questionnaire-9.

^c^DAIC-WOZ: Distress Analysis Interview Corpus/Wizard-of-Oz Dataset.

^d^PHQ-8: Patient Health Questionnaire-8.

^e^AVEC: Audio/Visual Emotion Challenge Dataset.

^f^BDI-II: Beck Depression Inventory.

^g^MODMA: Multi-modal Open Dataset for Mental Disorder Analysis.

Finally, the D3TEC dataset also stands out for its high-quality recording standards. Of all the public datasets on depression classification, D3TEC and MODMA (Multi-modal Open Dataset for Mental Disorder Analysis) are the only datasets that feature audios recorded to a professional quality standard, taking CD audio quality as the baseline (44.1 kHz, 16 bits) [11]. These are the only datasets that specify the microphone model used for recording as well.

Although this work is focused on audio analysis, it is important to note there are other biomarkers that can be used for automated depression classification. A very interesting research area is based on electroencephalography data analysis using machine learning techniques. A lot of progress has been made, achieving promising results. In the studies conducted by Soni et al [12,13], authors use electroencephalography to analyze brain signals for depression detection. They propose a method for automatic feature extraction by first constructing a graph where the nodes represent the subjects in the dataset and edge weights are obtained using the Euclidean distance, which reflects the relationship between them. The results show that the proposed method detects depression with an accuracy of 0.933. Although these are impressive results, our approach is different as we are focusing on voice analysis rather than electroencephalography signals analysis. We believe that creating a new dataset based on audio recordings is more feasible, low-cost, scalable, and ubiquitous compared to building an electroencephalography-based dataset.

### Problem Definition

Arguably, the most important requirement to build a high-performance deep learning model is to have a large amount of quality training data. Few depression datasets include voice recordings and even fewer are openly available. There are 4 main voice depression datasets available upon request. The most relevant of these is the DAIC-WOZ [10], collected in English; it features 189 recordings from the same number of participants. In addition, AVEC (Audio/Visual Emotion Challenge Dataset) 2013 [14] and AVEC 2014 [15], both featuring 84 German language speakers, contain 150 and 300 recordings, respectively. Finally, the MODMA [11] dataset, which contains data in Chinese, features 1508 recordings from 52 different subjects.

As noted by Wu et al [6] (2022), the scarce availability of datasets is one of the main roadblocks for research in voice depression classification, and currently available data have some important problems and limitations. We can point out 4 main issues:

1. Language barrier: datasets are available in very few languages. An English-spoken dataset should not be used in training if the intended purpose of the model is to identify depression in another language. To the best of our knowledge, there is no voice depression dataset in Spanish available for download.
2. Low audio quality standards: audio-quality recording standards have been neglected in most datasets. Almost none follow the basic CD quality standard of 44.1 kHz, which is the minimum required to capture the whole range of human hearing with no aliasing according to the Nyquist–Shannon theorem [16,17]. Besides that, no dataset specifies the characteristics of the room in which the recording took place or if all the recordings were collected in the same room. This might lead to a potential bias if recordings of both classes were done in different rooms (ie, recording participants with depression in a clinic and those with depression in an office or studio) as room acoustics have a significant impact on voice recordings [18]. Finally, most datasets do not report either the specific microphone used on the recordings or the signal flow (microphone, preamp, and analog-to-digital or digital-to-analog conversion) used to capture the audio, which directly affects the frequency response and sonic characteristics of the captured recording.
3. Class imbalance: as it is easier to find participants without depression, most datasets present a class imbalance. This means that both classes (depression and nondepression) have a different number of samples. This needs to be addressed during development to prevent the model from being biased toward the majority class [19,20].
4. Scarce smartphone research: most datasets have data collected in what can be inferred to be controlled environments, although the specific sonic characteristics of these environments are vaguely specified. Aiming to implement this technology in mobile apps, the effect of using a smartphone microphone instead of a professional one remains unknown. There is only 1 depression dataset that includes smartphone recordings called SH2, but it was collected privately by Sonde Health researchers, and it is not publicly available [21].

### Objectives

The present protocol gives guidelines for the collection of a new dataset that aims to address current issues in data availability for depression classification research, specifically: lack of language variety, low audio quality controls, and scarcity of data recorded with smartphone microphones. Although this work focuses on collecting data from Spanish speakers, this research protocol’s guidelines could be used to collect data in other languages as well.

The D3TEC dataset is designed with 3 main objectives in mind:

1. Spanish language: there is little language variety in current depression datasets. We focus on collecting data in Spanish, which has been an underrepresented language in voice depression classification.
2. High audio quality: audio is captured with studio-grade quality standards. This includes a low-noise environment, professional-grade recording equipment, a large diaphragm condenser microphone (Shure SM-27), and a high-quality recording format (44.1 kHz, 24 bits). In addition, potential sources of bias are controlled, including room acoustics.
3. Additional smartphone recordings: all professional recordings are also simultaneously captured by a smartphone microphone (iPhone SE2020). This provides data that can be used to evaluate the performance of deep learning models in depression classification when using smartphone recordings during training.

This data collection protocol is proposed by a multidisciplinary team that includes a professional audio engineer, 2 clinical psychologists, and 2 computer science researchers.

## Methods

### Participants

In deep learning, having a large dataset is desirable. However, as depression is a sensitive subject, it is not easy to include people with depression symptoms as research participants, thus depression datasets tend to be small [6]. We aimed at capturing recordings of at least 60 people, this number was established using other available depression datasets as a reference [11,14,15]. A total of 62 subjects are included in the final data collection. Data were collected in 2 different health centers, Tecnológico de Monterrey’s Centro de Bienestar Estudiantil (ITESM) and Centro Académico de Atención en Bienestar Integral (CAABI). The former gives mental health care to active ITESM students. The latter was established by the TEC Salud Foundation in San Gilberto, a low-income county in Santa Catarina, Nuevo León. Its goal is to give health care and psychological therapy at an affordable cost.

The inclusion criteria are the following: age between 18 and 65 years; live in Nuevo León, Mexico; enrolled in one of the health centers either as an active ITESM student or as an active patient at CAABI. Exclusion criteria are: engaged in illicit drug consumption in the past 2 weeks; adapting to a new psychiatric medicine that has been prescribed less than 1 month ago.

We established a relationship with each health center where patients are invited to participate in the voice recording session before or after their psychological appointment. An incentive of 200 MXN is offered to each participant as compensation for their time and participation in the study. This amount approximately equals US $12 in 2024.

### Data Collection

We collected 3 different kinds of data. The 2 most important ones are voice recordings and the depression screening questionnaire called PHQ-9 which is used to label the data [20]. In addition, we collected data on variables that could potentially affect the way a person speaks. The PHQ-9 scores encoded as binary values are the outcomes that are meant to be used as prediction labels in a machine learning model. It is important to note that the predictors in this case would only be the voice recordings; the additional demographic variables were collected only for control and data balance purposes.

Participants are asked to record their voices performing a variety of tasks prompted by a slideshow presentation. These include 3 categories: open questions, text fragments, and image descriptions as suggested by Wu et al (2022) [6]. Subjects are asked to aim for 30-second answers to each one of the 27 prompts. The recording tasks and time frame are established with the future goal of implementing the technology on a smartphone app. The prompts used on the slideshow presentation can be easily implemented on a mobile application, contrary to interactive interviews used on the DAIC-WOZ [10].

Each of the tasks has a different emotional valence. Positive valence refers to a prompt that makes the participant think of something positive. Neutral and negative valence evoke these emotions, respectively. An example of an open question used for each emotional valence is stated as follows:

1. What or who motivates you? Why? (Positive)
2. Describe your morning routine. (Neutral)
3. Which things make you angry? (Negative)

After the recording process, the participant is asked to fill out the PHQ-9 questionnaire. This is a depression screening test that includes 9 questions. Each question inquires about 1 symptom related to depression and the person must rate it on a scale from 0 to 3 according to how many times they have experienced that symptom in the last 2 weeks. Each audio is labeled with the obtained PHQ-9 score, which ranges from 0 to 27. A higher score indicates a higher presence of depression symptoms. Each audio is also labeled with a binary value of 0 (nondepressed class) when the PHQ-9 score is less than 10, and with a value of 1 (depressed class), when the PHQ-9 score is greater than or equal to 10.

The PHQ-9 cutoff score of 10 has been validated in numerous studies and is considered to effectively identify moderate to severe depression, ensuring those who need further mental health evaluation are identified while reducing false positives. It is important to note that the choice of cutoff score can affect the balance between missing cases of depression (false negatives) and incorrectly identifying individuals who do not have depression (false positives). Therefore, the choice of a cutoff score often depends on the screening’s goals and the context in which the screening tool is used [22,23].

Finally, we also collected some additional data. Although not intended to be used as a label or inclusion or exclusion criteria, we considered it important to collect data that could potentially affect the way a person speaks. Therefore, if a deep learning model trained using this dataset classifies data in an unexpected way, these variables can be analyzed to identify a potential cause of bias. The additional control variables are shown in Table 2.

**Table 2:** Additional control data.

Variable	Value	Reason to collect
Age (years)	18-65	Biological aging of vocal tract
Sex (biological)	Male or female	Biological differences on vocal tract
Place of residence	City, State, Country	Influence on accent
Place of birth	City, State, Country	Influence on accent
Social class	Working, middle, or upper^a^ class	Influence on accent
Data collection center	ITESM^b^ or CAABI^c^	Potential influence on accent. Influence on room acoustics
Medicine	Current medication or none	Potential influence on speech or mood
Physical health condition	Current health condition or none	Potential influence on speech
Mental health condition	Current diagnosis or not available	If a formal diagnosis exists, it is relevant

^a^Social class data were assigned according to average household monthly income in Mexican pesos. We used 13,000 MXN (~US $758) for the working class, 23,000 MXN (~US $1341) for the middle class, and 78,000 MXN (~US $4548) for the upper class which is the average for each social class in Mexico according to data provided by Instituto Nacional de Estadística y Geografía [24].

^b^ITESM: Tecnológico de Monterrey’s Centro de Bienestar Estudiantil.

^c^CAABI: Centro Académico de Atención en Bienestar Integral.

### Audio Recordings and Quality Standards

A total of 27 audios are recorded by each subject. These audios are collected simultaneously by a professional microphone and a smartphone microphone. This is meant to be used by researchers to evaluate how audio quality affects deep learning–based models’ performance in depression classification.

The purpose of high-quality recordings is to collect audio with the highest amount of information possible. Even if most humans cannot differentiate between uncompressed (WAV and AIFF) and compressed (MP3, OGG, FLAC, etc) audio formats, there is a significant difference in the amount of information present in each format. A file size comparison is shown in Table 3. These sizes are calculated by transforming a 44.1 kHz, 24-bit WAV into different formats using iZotope RX format and Cloudconvert. It clearly shows how a 44.1 kHz, 24-bit WAV file can have up to 15 times more data than a compressed 128 kbps AAC file.

**Table 3:** File size of 1-minute WAV files recorded with different sample rate and bit depth parameters before and after file compression.

Sample rate	Bit depth	File size (uncompressed)	File size (mp3, 320 kbps)	File size (AAC 128 kbps)
44.1 kHz	24 bits	15.9 MB^q^	2.4 MB	0.96 MB
44.1 kHz	16 bits	10.6 MB	2.4 MB	0.96 MB
41 kHz	16 bits	9.8 MB^b^	2.4 MB	0.96 MB^c^
16 kHz	24 bits	5.8 MB	1.2 MB	0.96 MB
16 kHz	16 bits	3.8 MB^d^	1.2 MB	0.96 MB

^a^MODMA (Multi-modal Open Dataset for Mental Disorder Analysis dataset).

^b^AVEC (Audio/Visual Emotion Challenge Dataset) 2013 dataset.

^c^AVEC 2014 dataset.

^d^DAIC-WOZ (Distress Analysis Interview Corpus/Wizard-of-Oz Dataset) dataset.

Currently, no study compares the effect of audio quality on the performance of deep learning–based models for depression classification. The only reason to capture audio with lower quality is to save digital space, but this decision should not be taken if the effect of reducing quality is unknown. Higher-quality audio can be converted into lower-quality formats; therefore, capturing audio to a high-quality standard is desirable, as this renders audio with more information and allows researchers to change the audio format and run experiments considering different audio qualities.

The high-quality audio section of the D3TEC dataset was recorded with a high audio quality standard. A sample rate of 44.1 kHz is established since it is the minimum needed to represent the whole audible frequency spectrum while avoiding aliasing [17]. Although using 16 bits is standard CD quality, the higher bit-depth standard of 24 bits is chosen since it allows for more dynamic range. The complete recording parameters of the D3TEC dataset are shown in Textbox 1.

**Hardware, software, and recording parameters for the high-quality audios of the Dataset for the Detection of Depression of Tecnológico de Monterrey dataset.**

**Recording parameters**

- Sample rate: 44.1 kHz
- Bit depth: 24 bits
- Stereo or mono: mono

**Hardware**

- Microphone: Shure SM27 (large diaphragm condenser)
- Analog-to-digital interface: AVID Fast track duo

**Software**

- DAW: Logic Pro X

**Other**

- Mouth distance from microphone at 20 cm, approximately.
- No pop filter used.
- All recordings share the same gain configuration. The interface’s gain knob is marked at 6/10. Gain level is established moderately to avoid clipping considering that voice volume varies from person to person.
- RT60 time on both recording centers is less than 1 second.

The purpose of recordings using a smartphone is for researchers to be able to evaluate the performance of deep learning–based models when a smartphone is used instead of a professional microphone. The goal of these recordings is to make them representative of a real case scenario in which a person records their voice with their smartphone in a quiet room. All audios are recorded on an iPhone SE2020 using the Apple Voice Memos application with a sample rate of 48 kHz in the compressed format M4A, which is the default audio format of the app. The smartphone is placed on a desk in front of the subject at approximately the same distance the professional microphone is placed. Audios are afterward converted to a WAV format of 44.1 kHz, 24 bits for processing; however, converting low-quality audio to a higher-quality format does not add new information. Therefore, these audios should be considered M4A, even if converted to WAV for practical purposes.

For binary classification, a neural network-based model must be able to correctly classify data into one of 2 classes: depression and no-depression. Broadly speaking, the model accomplishes this by learning common patterns present in each class that make both classes different. A bias occurs when the model classifies the data, taking as a common pattern something that the developer did not intend or anticipate. For example, if a model is meant to train in and classify a dataset of circles and squares, but all circles are red and all squares are blue, one cannot assume that the model learned the pattern that separates a circle from a square. It could be the case that the model learned to classify according to color, not shape.

The same applies to audio. The voice of the person is not the only factor at play, many other factors affect the way an audio recording sounds. For example, room acoustics, microphone, audio interface, preamp, sample rate, bit-depth, audio format, and gain staging. Say for example that a data collector decided to record participants with depression in a clinic with concrete walls and people without depression in a studio with wooden walls. A model trained with these data might present a bias caused by the room acoustics, incorrectly classifying voices recorded in concrete-walled rooms as depressed.

For the D3TEC dataset, we undertook several measures to avoid potential bias in audio recordings. We used the same room at each data collection center with the same microphone placement. All audios are recorded with the same 2 microphones simultaneously, Shure SM-27 and iPhone SE 2020. For every recording, we used the same audio interface (AVID Fast Track Duo) and the same channel, preamp, and gain level. Finally, we used Logic Pro X software for professional audio and the Voice Memos App for smartphone audio.

### Ethical Considerations

This research protocol has been approved by the Institutional Research Ethics Committee of Tecnológico de Monterrey (protocol code CA EIC-2407-03; Multimedia Appendix 1). All participants provided written informed consent after being briefed about the study’s goals and were informed about the ability to opt out of the process at any stage of the protocol (Multimedia Appendix 2). All audio recordings and collected data were anonymized and deidentified to comply with ethical and personal data privacy requirements. An incentive equivalent to US $12 in 2024 was offered to each participant as compensation for their time and participation in the study. The ethics committee approval and informed consent forms can be found in the Multimedia Appendices 1 and 2.

## Results

This section presents the final collected dataset. The main demographic information of the D3TEC dataset is displayed in Tables S1 and S2 in Multimedia Appendix 3, which contain the suggested training and testing set, respectively. The full dataset including audio and every collected variable can be found in IEEE DataPort [25].

The variables included in Tables S1 and S2 in Multimedia Appendix 3 are described as follows. ID refers to a unique code given to each participant, which is used instead of their name for anonymization purposes. PHQ-9 is the depression screening score as obtained in the PHQ-9 questionnaire. A Boolean value, PHQ-9 binary for depression, is included using a score of 10 as the cutoff value. Demographic information is included such as age, biological sex, and place of residence. Social class is recorded based on the monthly household income of each participant and classified into lower, middle, or upper class using the INEGI scale for social class quantification [24]. Finally, the medicine variable indicates if the participant was taking medication at the time of the recording session. These include any kind of medication, psychiatric and nonpsychiatric.

The D3TEC dataset includes audio recordings from 62 participants, recording 27 audios each, which equals 1674 voice recordings. The dataset is split into suggested training and test sets. The training set has 79% of the dataset while the remaining 21% is designated as the test set. However, researchers can decide on alternative approaches for splitting the data according to their needs.

## Discussion

### Overview

The protocol to create the D3TEC dataset was carefully designed to address the current limitations of data availability for deep learning research in voice depression classification, which include limited language diversity, low audio quality standards, class imbalance, and scarcity of audio data collected through smartphones. We undertook the following actions during the data-gathering process, to guarantee the quality of the resulting dataset. First, the D3TEC dataset contains audio samples in Spanish, a language that has been largely underrepresented in voice depression classification. Second, every audio was collected with a professional, studio-grade microphone in a noiseless environment. Moreover, several measures were taken to avoid potential bias by controlling room acoustics and recording parameters. Third, in our dataset, the nondepressed class is 53% and the depressed class is 47%, indicative of a reduced class imbalance, especially when compared to the DAIC-WOZ dataset, where the class imbalance is a concern [10]. Finally, alongside the professional microphone, every audio was simultaneously recorded with a smartphone, providing data that enables researchers to evaluate the feasibility of deploying voice depression classification models in mobile apps. It is worth mentioning that the D3TEC dataset is publicly available and can be downloaded through IEEE DataPort (Multimedia Appendix 3).

As noted by Wu et al [6], one of the most important roadblocks preventing the development of automated depression recognition technology is the scarcity of data collected for this purpose. Our work is focused not only on recording voice data of individuals with depression, but also on providing data with characteristics that are not present in currently available datasets. Therefore, the D3TEC dataset is expected to open new research paths in automated depression classification. First, the inclusion of recordings in Spanish enables the development of robust deep learning models that can identify depression in Spanish-speaking populations; this language has been largely underrepresented in currently available datasets including DAIC-WOZ, MODMA, and AVEC [10,11,14]. Second, the inclusion of recordings in the dataset, captured simultaneously with professional and smartphone microphones, allows for a comparative analysis of device impact on model performance. These dual-format data are expected to enable research focused on the development of deep learning depression classification models that can be deployed in mobile apps. Therefore, the technology will be scalable, available, and usable in real-world applications. Third, the high audio-quality standards of the dataset, which include controlled room acoustics and recording parameters, are anticipated to minimize biases often present in voice data. It is worth noting that the most popular datasets shown in Table 1, present lower audio quality standards than the D3TEC; only MODMA was recorded with comparable recording quality [11]. Finally, the inclusion of additional control variables allows for potential demographic-induced bias identification. All these attributes are expected to significantly reduce overfitting problems when developing deep learning classification models for automated depression identification.

We are providing the research community with a new dataset developed with high-quality standards, regarding room ambiance and recording parameters. The selection of the Spanish language alleviates the current shortage of datasets in this language, considering that Spanish is the second most spoken native language in the world, with almost 500 million people [26]. The implication of this is a higher coverage of the number of people that potentially will benefit from any health-monitoring application resulting from forthcoming research. We hypothesize that new deep learning models trained and tested on this dataset will benefit from a reduced bias and overfitting; therefore, performance metrics, such as *F*_1_-score, precision, and recall will be more reliable. In addition, our goal is to assess the effect of audio quality on the performance of deep learning–based models for depression classification. The number of participants in the dataset, 62, is very competitive compared to the other datasets found in the literature. The resulting balance between classes, depressed/not-depressed, is also a very positive property. We are currently working on the development of deep learning models trained and tested on the D3TEC dataset to prove the validity of our hypothesis.

### Limitations

In the following paragraphs, we discuss the limitations and strengths of the presented dataset and potential issues that should be considered when using it. The main limitations discussed are label quality, the potential influence of prescribed medication, and biological sex imbalance.

The most important information for each data entry is whether the audio comes from a participant with or without depression. In this dataset, that ground truth is based on the PHQ-9 questionnaire, which is a screening tool that has proven valuable in identifying depression and can be self-administered [23]. Although it performs well when compared to other questionnaires, the PHQ-9 is only a screening tool and it cannot be used on its own to determine if a subject is, in fact, depressed. That diagnosis can only be given by a clinical psychologist, which usually takes several sessions. Although the original idea was to label subjects using a clinical diagnosis, this proved to be impractical, expensive, and time-consuming. It would take at least 3 sessions to diagnose a potential participant in each of the health centers used for data collection. Thus, to collect more recordings in a shorter time frame, we chose to label them with the PHQ-9. It is important to recall that this label is not as good as a clinical diagnosis.

Regarding the potential influence of prescribed medication, some of the participants who scored high on the PHQ-9 questionnaire were also taking antidepressant medication, such as sertraline and fluoxetine. These medications are meant to reduce depression symptoms. This could have a negative impact on data collection since a person’s score on the PHQ-9 could be affected by the medication. Other datasets took their participants off medication 2 weeks before recording; however, this approach represents a medical risk for the participant’s emotional well-being, therefore we decided not to follow it [11]. In our case, we decided to collect the variable, hence it can be traced back, in case we suspect bias due to medication.

The dataset currently presents a slight class imbalance. The nondepressed class is 53% and the depressed class is 47%. The presence of class imbalance is common in depression datasets, but this needs to be considered when using the data for deep learning research [6,19]. On top of the class imbalance, the dataset presents a biological sex imbalance: 58% of participants are female and 42% male. This imbalance must be considered when splitting the data into training and testing sets for deep learning research.

Although we worked with 2 different data collection centers, most audio recordings were made at ITESM and only 9 participants were recorded at CAABI. When working with the dataset, removing the 9 audios from CAABI for deep learning experimentation might be beneficial in some applications, as this removes an additional recording space and demographic population that could introduce noise in the results. The decision whether to use the CAABI recordings or not must be taken by each researcher using this dataset according to the goals of their research. For identification purposes, a CAABI participant ID starts with 0 and an ITESM participant ID starts with 1.

### Conclusions

This research outlines a thorough protocol for creating a dataset of depression voice recordings for deep learning research, with 3 novel characteristics: it has a high audio quality standard, it features Spanish speakers, and it collects data simultaneously using professional and mobile devices; hence, overcoming main issues on current voice datasets for depression classification.

Some of the research areas enabled by this new dataset are binary and multiclass depression classification of Spanish speakers. Also, it allows for a clear assessment of the impact of audio and microphone quality on the performance of depression classification models. This is of great relevance, since evaluating if there is a statistically significant difference in training these models with mobile collected data, opens the door for a lower cost, more flexible and scalable data-gathering process. Furthermore, it will make wearable-based applications more feasible.

Prior research, mainly performed on the DAIC-WOZ dataset, shows that the computational analysis of voice recordings can be a useful way of identifying depression, with performance as high as an *F*_1_-score of 85% in the best algorithms. The development of new deep learning models trained and tested on datasets created with high-quality standards paves the path for new practical and research applications in voice depression classification, assuring better performance metrics, such as *F*_1_-score, precision, and recall.

As the process of collecting new datasets continues, the prospects of creating an objective, accessible, and noninvasive tool for screening depression become increasingly tangible. Future research is meant to expand upon these foundations, exploring the integration of this technology into clinical practices and the potential for its application across different languages and cultural contexts.

## Appendix

Multimedia Appendix 1 Ethics committee approval.

Multimedia Appendix 2 Informed consent form.

Multimedia Appendix 3 Tables describing the D3TEC (Dataset for Depression Detection of Tecnológico de Monterrey) dataset structure as published in IEEE Dataport.

## Abbreviations

AVEC: Audio/Visual Emotion Challenge Dataset
BDI-II: Beck Depression Inventory
CAABI: Centro Académico de Atención en Bienestar Integral
D3TEC: TEC de Monterrey’s Depression Detection Dataset
DAIC-WOZ: Distress Analysis Interview Corpus/Wizard-of-Oz Dataset
ITESM: Tecnológico de Monterrey
MODMA: Multi-modal Open Dataset for Mental Disorder Analysis
PHQ-9: Patient Health Questionnaire
