# Supplementary material for: Data Collection for Automatic Depression Identification in Spanish Speakers Using Deep Learning Algorithms: Protocol for a Case-Control Study
Source: JMIR Res Protoc. 2025 Jul 31;14:e60439. doi: 10.2196/60439 (PMC12355134; doi:10.2196/60439)
Supplement: Multimedia Appendix 1 [file resprot_v14i1e60439_app1.pdf]

Monterrey, Nuevo León a 12 de septiembre del 2024.

Carta de Aprobación al Protocolo Evaluado
Comité Institucional de Ética en la Investigación

Estimado Investigador Luis Ángel Trejo Rodríguez,

Por medio de esta carta le informamos a Usted que el Comité Institucional de Ética en la Investigación (CIEI) del Tecnológico de Monterrey llevó a cabo la revisión al protocolo de investigación titulado: **“DLDEP: Deep Learning para reconocimiento de la depresión en la voz, utilizando grabaciones de teléfono celular en idioma español.”**

El resultado de la autoevaluación realizada por el equipo de investigación estima que el protocolo cumple con los requisitos éticos y representa riesgo bajo, las y los miembros del comité coinciden con esta valoración en sesión ordinaria, por lo que otorgan el carácter de:

APROBADO

Los investigadores son responsables de la metodología que se utiliza en la investigación. El código de seguimiento de este protocolo es: **CA EIC-2407-03.** Y su vigencia de la aprobación será al 12 de septiembre del 2025, sujeto a que no haya modificaciones en el protocolo durante la implementación. Como parte de su compromiso deberá entregar un reporte breve del proyecto, a más tardar 30 días al término de este.

Atentamente,

Mario Moisés Alvarez
Representante de la Escuela de Ingeniería y Ciencias
Comité Institucional de Ética en la Investigación
Tecnológico de Monterrey
