# Supplementary material for: Data Collection for Automatic Depression Identification in Spanish Speakers Using Deep Learning Algorithms: Protocol for a Case-Control Study
Source: JMIR Res Protoc. 2025 Jul 31;14:e60439. doi: 10.2196/60439 (PMC12355134; doi:10.2196/60439)
Supplement: Multimedia Appendix 2 [file resprot_v14i1e60439_app2.pdf]

Consentimiento Informado

Introducción

Queremos hacerle una invitación a participar voluntariamente en un proyecto de investigación. Este proyecto tiene como objetivo ***"Expandir el estado del arte actual en identificación de depresión en voz, al desarrollar y evaluar el primer dataset de depresión que contiene grabaciones en español. Y al profundizar la investigación actual en grabaciones de teléfonos celulares al compararlas con grabaciones profesionales.*** "

Antes de que usted acepte participar en este estudio, le pedimos su **consentimiento informado**. Es de suma importancia que lo lea cuidadosamente el propósito y objetivos del estudio. Si usted tiene preguntas puede hacerlas directamente al investigadorresponsable quien le ayudarán a resolver cualquier inquietud. Su decisión de participar es voluntaria, lo que significa que usted es totalmente libre de ingresar a o no en el estudio. Podrá retirar su consentimiento en cualquier momento y sin tener que explicar las razones.

Propósito del Estudio

A través de este documento queremos hacerle una invitación a participar voluntariamente en un estudio de investigación. Tiene como **objetivo crear una colección de grabaciones de voz de personas con y sin depresión que pueda ser utilizada para investigar si es posible detectar sígnos de depresión en la voz humana a través de la computación.**

Se le ha pedido participar en este estudio porque usted es mayor de edad, residente de Nuevo León y entra en alguno de estos dos grupos: (1) Tiene síntomas de depresión. (2) No tiene síntomas de depresión, pero comparte características demográficas (edad, sexo) con alguien que sí.

En el estudio participarán al menos 60 personas, pero el mayor número de participantes posible es ideal.

¿Qué sucederá durante el estudio de Investigación?

Se le pedirá asistir a una única sesión de grabación de **15-20min** que consta de 2 pasos:

1. **Documentación:** debe llenar una serie de documentos que incluyen: (1) El presente consentimiento informado, (2) cuestionario PHQ-9, (3) cuestionario de datos demográficos (edad, sexo, residencia, entre otros...).
2. **Grabación:** Participar de una sesión de grabación de voz respondiendo a las siguientes tareas: Respuesta a preguntas abiertas, lectura de fragmentos de texto, descripción de imágenes.

En retribución a su tiempo y dedicación en este estudio se le ofrecerá una compensación de 200 pesos mexicanos, entregados en un sobre.

Es importante que considere que este set de grabaciones será de libre acceso para otros investigadores. Sin embargo los registros que lo identifican se mantendrán en forma confidencial y, hasta donde lo permitan las leyes y/o regulaciones aplicables, no se harán del conocimiento público. Si los resultados del estudio se publican, su identidad se mantendrá confidencial. Usted no será identificado en ninguno de los reportes o publicaciones que resulten de este estudio. El cuidado confidencial de los datos y procedimiento del estudio es responsabilidad del equipo de investigación. **Tanto el Centro de Académico de Atención en Bienestar Integral (CAABI) y el departamento de Bienestar Estudiantil quedan excluidos de toda responsabilidad.**

Este Consentimiento ha sido revisado por el *Comité de Ética en Investigación del Instituto Tecnológico y de Estudios Superiores de Monterrey.* Si tiene alguna preocupación o queja acerca de este estudio o sobre cómo se está realizando, o alguna pregunta con respecto a sus derechos, usted puede comunicarse al (01) 81 88882107 o al correo electrónico A00819817@tec.mx

Yo ______________________________________, acepto participar en este estudio de investigación, soy mayor de edad y recibí una copia de este consentimiento informado. Soy consciente de que puedo retirarme del estudio en cualquier momento y solicitar que mis datos sean eliminados si así lo deseo.

____ ____

Firma del Participante Luis F. Brenes – Investigador a cargo del estudio
