# Supplementary material for: Data Collection for Automatic Depression Identification in Spanish Speakers Using Deep Learning Algorithms: Protocol for a Case-Control Study
Source: JMIR Res Protoc. 2025 Jul 31;14:e60439. doi: 10.2196/60439 (PMC12355134; doi:10.2196/60439)
Supplement: Multimedia Appendix 3 [file resprot_v14i1e60439_app3.docx]

**Table S1.** D3TEC Dataset (training set 79%).

| ID | PHQ-9 | Sex | PHQ- 9 (Binary) | Age | Place of Residence | Social Class | Medicine |
| --- | --- | --- | --- | --- | --- | --- | --- |
| 116 | 1 | Male | False | 23 | N.L., Mexico | Upper | False |
| 148 | 5 | Male | False | 18 | N.L., Mexico | Upper | False |
| 112 | 5 | Female | False | 18 | N.L., Mexico | Upper | True |
| 152 | 5 | Male | False | 22 | N.L., Mexico | Upper | False |
| 005 | 7 | Female | False | 40 | N.L., Mexico | Middle | True |
| 149 | 12 | Male | True | 19 | N.L., Mexico | Upper | False |
| 003 | 3 | Female | False | 62 | N.L., Mexico | Upper | True |
| 128 | 11 | Female | True | 22 | N.L., Mexico | Upper | True |
| 138 | 14 | Female | True | 19 | N.L., Mexico | Working | False |
| 009 | 21 | Female | True | 41 | N.L., Mexico | Working | False |
| 134 | 6 | Male | False | 22 | N.L., Mexico | Upper | False |
| 106 | 24 | Female | True | 20 | N.L., Mexico | Middle | True |
| 142 | 4 | Female | False | 20 | N.L., Mexico | Middle | True |
| 139 | 8 | Male | False | 22 | N.L., Mexico | Upper | True |
| 114 | 23 | Female | True | 20 | N.L., Mexico | Middle | True |
| 146 | 10 | Female | True | 20 | N.L., Mexico | Middle | False |
| 151 | 4 | Male | False | 20 | N.L., Mexico | Upper | False |
| 118 | 8 | Male | False | 20 | N.L., Mexico | Middle | False |
| 130 | 5 | Female | False | 20 | N.L., Mexico | Upper | True |
| 135 | 9 | Male | False | 22 | N.L., Mexico | Upper | False |
| 004 | 13 | Female | True | 48 | N.L., Mexico | Working | False |
| 137 | 10 | Female | True | 21 | N.L., Mexico | Middle | False |
| 143 | 11 | Female | True | 21 | N.L., Mexico | Upper | False |
| 133 | 11 | Female | True | 19 | N.L., Mexico | Upper | False |
| 140 | 4 | Male | False | 21 | N.L., Mexico | Middle | False |
| 147 | 3 | Male | False | 23 | N.L., Mexico | Upper | False |
| 153 | 19 | Male | True | 20 | N.L., Mexico | Middle | False |
| 119 | 1 | Male | False | 18 | N.L., Mexico | Upper | False |
| 121 | 13 | Male | True | 24 | N.L., Mexico | Middle | True |
| 008 | 12 | Female | True | 49 | N.L., Mexico | Middle | False |
| 107 | 5 | Male | False | 22 | N.L., Mexico | Upper | False |
| 132 | 20 | Male | True | 20 | N.L., Mexico | Upper | False |
| 103 | 15 | Male | True | 22 | N.L., Mexico | Upper | False |
| 136 | 19 | Male | True | 20 | N.L., Mexico | Upper | False |
| 117 | 7 | Male | False | 20 | N.L., Mexico | N/A | False |
| 129 | 2 | Male | False | 19 | N.L., Mexico | Upper | False |
| 123 | 10 | Male | True | 19 | N.L., Mexico | Upper | False |
| 122 | 15 | Male | True | 20 | N.L., Mexico | Upper | False |
| 131 | 13 | Male | True | 19 | N.L., Mexico | Upper | False |
| 102 | 6 | Female | False | 20 | N.L., Mexico | Working | False |
| 145 | 5 | Female | False | 21 | N.L., Mexico | Upper | False |

**Table S2.** D3TEC Dataset (test set 21%).

| ID | PHQ-9 | Sex | PHQ- 9 (Binary) | Age | Place of Residence | Social Class | Medicine |
| --- | --- | --- | --- | --- | --- | --- | --- |
| 111 | 16 | Female | True | 19 | N.L., Mexico | Upper | True |
| 115 | 2 | Male | False | 23 | N.L., Mexico | Upper | False |
| 108 | 14 | Female | True | 24 | N.L., Mexico | Upper | False |
| 120 | 12 | Female | True | 22 | N.L., Mexico | Middle | True |
| 113 | 12 | Female | True | 19 | N.L., Mexico | Middle | True |
| 124 | 11 | Female | True | 21 | N.L., Mexico | Upper | False |
| 125 | 10 | Female | True | 23 | N.L., Mexico | Middle | False |
| 126 | 2 | Female | False | 21 | N.L., Mexico | Middle | False |
| 144 | 3 | Female | False | 22 | N.L., Mexico | Middle | False |
| 141 | 2 | Male | False | 19 | N.L., Mexico | Upper | True |
| 127 | 9 | Male | False | 22 | N.L., Mexico | Upper | False |
